# Comment on: Access and adequacy of antenatal care in during two phases of the COVID-19 pandemic

**DOI:** 10.61622/rbgo/2025rbgo12

**Published:** 2025-03-17

**Authors:** Amnuay Kleebayoon, Viroj Wiwanitkit

**Affiliations:** 1 Private Academic Consultant Samraong Cambodia Private Academic Consultant, Samraong, Cambodia.; 2 Saveetha Institute of Medical and Technical Sciences Saveetha University Saveetha Dental College and Hospitals Department of Research Analytics Chennai India Department of Research Analytics, Saveetha Dental College and Hospitals, Saveetha Institute of Medical and Technical Sciences Saveetha University, Chennai, India.

The publication on "Access and adequacy of antenatal care in a city in Brazil during two phases of the COVID-19 pandemic."^(1)^ Is an interesting issue. This study investigated antenatal care consumption and appropriateness among postpartum caregivers at Florianópolis Hospital from 2020 to 2022, with an emphasis on socio-demographic characteristics and antenatal care. Although this study gives significant insights, there are some methodological problems and areas for improvement. The observational cross-sectional design captures only one point in time, restricting the capacity to demonstrate causal linkages. A longitudinal or cohort approach may provide more detailed insights into patterns and changes over time. Furthermore, while antenatal medical records and pamphlets are commonly used as primary data sources, documentation discrepancies and recollection bias can arise, particularly when data points are acquired from historical records. It would be beneficial to include more thorough data sources or analysis tools.

Although the Carvalho and Novaes^(2)^ index is an intriguing method for determining the adequacy of antenatal treatment, its generalizability should be questioned. Can it be used to a wide range of populations and address all areas of healthcare quality? Although we address major barriers, such as the impact of sickness, these considerations may be incomplete. Other barriers, such as socioeconomic status, education level, and cultural characteristics, may have a substantial impact on access and appropriateness of care, but have not been thoroughly investigated. Future study should include a broader range of variables. This encompasses the various aspects that may influence individual healthcare experiences.

This study's focus on adequacy of care and access to healthcare is one of its strengths, but it also raises fundamental problems concerning adequacy measurement. The adequacy rate was 48.6% in 2020, compared to 69.1% in 2022, indicating an improvement, however the causes for this shift remain unknown. Are these developments the result of systematic advances in healthcare delivery, or are these figures influenced by other factors such as changes in population health requirements or healthcare priorities? These issues require more investigation through qualitative interviews and focus groups in order to better understand the underlying causes and enhance the appropriateness of care.

Finally, the findings of this study suggest various areas for future research. Given the tremendous influence that the COVID-19 pandemic has had on healthcare access, future research should look into how pandemic-specific policies, such as the implementation of telemedicine and changes in clinic operations, have affected prenatal care. New approaches could include investigating the views of healthcare providers and patients to better understand the problems clinicians encounter in providing quality treatment during the crisis. Expanding the study to encompass a broader range of health care settings would provide a more complete picture of prenatal care practices and access in different locations and systems.
