# Supplementary material for: Comment on: Access and adequacy of antenatal care in during two phases of the COVID-19 pandemic
Source: Rev Bras Ginecol Obstet. 2025 Mar 17;47:e-rbgo12. doi: 10.61622/rbgo/2025rbgo12 (PMC12002718; doi:10.61622/rbgo/2025rbgo12)
Supplement: Related texts

# Author's reply to comment on: Access and adequacy of antenatal care in during two phases of the COVID-19 pandemic

**Authors:** Nicole Zazula Beatrici, Roxana Knobel, Mariana Schmidt Vieira, Iago Felipe Alexandrini, Alberto Trapani, Carla Betina Andreucci

**Affiliations:** 1 Universidade Federal de Santa Catarina Tocogynecology Department Florianópolis SC Brazil Tocogynecology Department, Universidade Federal de Santa Catarina, Florianópolis, SC, Brazil.; 2 Universidade Federal de São Carlos São Carlos SP Brazil Universidade Federal de São Carlos, São Carlos, SP, Brazil.

We appreciate your comments on our publication "Access and Adequacy of Antenatal Care in a City in Brazil during Two Phases of the COVID-19 Pandemic"^(1)^ and fully agree with your observations, which greatly enrich the discussion around the study. We concur that an observational study design has limited power to establish causal relationships, and we recognize this as a limitation of the methodology employed. While this approach captures valuable cross-sectional data, it lacks the depth that longitudinal or cohort studies could provide in identifying dynamic patterns or causal linkages. As you highlighted, we also acknowledge the potential issues related to prenatal records, which may reflect a broader reality in the country.^(2)^ Despite the risk of discrepancies or missing data, we consider these flaws essential findings. In the current state healthcare services organization, hospital teams primarily rely on these records to care for laboring patients. Nevertheless, we agree that specific research is necessary to evaluate the quality of these records and their impact on the care provided. We take this opportunity to emphasize the importance of promoting the electronic integration of prenatal data with hospital care services. Regarding the sample size and potential selection biases, we agree with your observations and have highlighted these issues as limitations in the article. We recognize these as inherent challenges in studies of this nature that require careful attention.

Although the Carvalho and Novaes^(3)^ index is exclusively quantitative, we used it to allow comparisons with other studies and, primarily, with data provided by the Brazilian Unified Health System's information system.^(4)^ While we acknowledge its limitations, we found it to be a useful tool to ensure consistency and comparability within the scope of this research. The Carvalho and Novaes^(3)^ index also has a high positive predictive value on adequate antenatal care access and good levels of adjusted agreement with other antenatal assessment indices.^(5)^

Finally, we fully agree that this study raises numerous important questions, including those proposed in your letter. These questions require further investigation using diverse methodological approaches to provide comprehensive answers.

## References

[1] Beatrici NZ, Knobel R, Vieira MS, Alexandrini IF, Trapani A, Andreucci CB (2024). Access and adequacy of antenatal care in a city in Brazil during two phases of the COVID-19 pandemic. Rev Bras Ginecol Obstet.

[2] Camargos LF, Lemos PL, Martins EF, Felisbino-Mendes MS (2020). Avaliação da qualidade dos registros de cartões de pré-natal de mulheres urbanas. Esc Anna Nery.

[3] Carvalho DS, Novaes HM (2004). Avaliação da implantação de programa de atenção pré-natal no Município de Curitiba, Paraná, Brasil: estudo em coorte de primigestas. Cad Saúde Pública.

[4] Ministério da Saúde (2023). Secretaria de Vigilância em Saúde e Ambiente.

[5] Tomasi E, Fernandes PA, Fischer T, Siqueira FC, Silveira DS, Thumé E (2017). Qualidade da atenção pré-natal na rede básica de saúde do Brasil: indicadores e desigualdades sociais. Cad Saúde Pública.
